# Antenatal care as a means to increase participation in the continuum of maternal and child healthcare: an analysis of the poorest regions of four Mesoamérican countries

**DOI:** 10.1186/s12884-019-2207-9

**Published:** 2019-02-12

**Authors:** Claire R. McNellan, Emily Dansereau, Marielle C. G. Wallace, Danny V. Colombara, Erin B. Palmisano, Casey K. Johanns, Alexandra Schaefer, Diego Ríos-Zertuche, Paola Zúñiga-Brenes, Bernardo Hernandez, Emma Iriarte, Ali H. Mokdad

**Affiliations:** 1 Institute for Health Metrics and Evaluation, 2301 5th Ave, Suite, Seattle, WA 600 USA; 2 Salud Mesoamérica Initiative/Inter-American Development Bank, Calle 50, Edificio Tower Financial Center (Towerbank), Piso 23, Panamá city, Panamá; 3 University of Washington Department of Health Metrics Sciences and Department of Global Health, 1510 San Juan Rd, Seattle, WA 98195 USA

**Keywords:** Antenatal care, Prenatal care, Maternal care, Salud Mesoamérica initiative, Skilled ANC, Household surveys

## Abstract

**Background:**

Antenatal care (ANC) is a means to identify high-risk pregnancies and educate women so that they might experience a healthier delivery and outcome. There is a lack of evidence about whether receipt of ANC is an effective strategy for keeping women in the system so they partake in other maternal and child interventions, particularly for poor women. The present analysis examines whether ANC uptake is associated with other maternal and child health behaviors in poor mothers in Guatemala, Honduras, Nicaragua, and Mexico (Chiapas).

**Methods:**

We conducted a cross-sectional survey of women regarding their uptake of ANC for their most recent delivery in the last two years and their uptake of selected services and healthy behaviors along a continuity of maternal and child healthcare. We conducted logistic regressions on a sample of 4844 births, controlling for demographic, household, and maternal characteristics to understand the relationship between uptake of ANC and later participation in the continuum of care.

**Results:**

Uptake of four ANC visits varied by country from 17.0% uptake in Guatemala to 81.4% in Nicaragua. In all countries but Nicaragua, ANC was significantly associated with in-facility delivery (IFD) (Guatemala odds ratio [OR] = 5.28 [95% confidence interval [CI] 3.62–7.69]; Mexico OR = 5.00 [95% CI: 3.41–7.32]; Honduras OR = 2.60 [95% CI: 1.42–4.78]) and postnatal care (Guatemala OR = 4.82 [95% CI: 3.21–7.23]; Mexico OR = 4.02 [95% CI: 2.77–5.82]; Honduras OR = 2.14 [95% CI: 1.26–3.64]), but did not appear to have any positive relationship with exclusive breastfeeding habits or family planning methods, which may be more strongly determined by cultural influences.

**Conclusions:**

Our results demonstrate that uptake of the WHO-recommended four ANC visits has limited effectiveness on uptake of services in some poor populations in Mesoamérica. Our study highlights the need for continued and varied efforts in these populations to increase both the uptake and the effectiveness of ANC in encouraging positive and lasting effects on women’s uptake of health care services.

## Background

Antenatal care (ANC) has repeatedly been shown to reduce neonatal deaths via identification of high-risk pregnancies [1–6]. Current WHO guidelines, however, state an additional underlying benefit: women’s uptake of ANC by a medical professional reduces dropout from the continuum of maternal and reproductive healthcare [7]. ANC plays a central role in the continuum of care, a critical framework for understanding the continuity between maternal, newborn, and child health. ANC is an opportunity for skilled professionals to educate and engage with women about how and why to deliver in a facility (in-facility delivery, IFD), the benefits of exclusive breastfeeding, where and when to return for postpartum and postnatal care, and the availability of modern family planning methods [1, 8]. Additionally, women who have a positive experience during their ANC visits may be more likely to bring their children back to health facilities for services such as vaccinations and nutritional supplements [2]. While this logic is intuitive and optimistic, little evidence exists to confirm that receipt of ANC effectively keeps women in the health care system, particularly poor populations [3, 8–10]. Given that poor women are often the least connected to health services due to long travel distances, lack of access to transportation or insurance, poor health literacy, financial constraints, or cultural beliefs, it is particularly important to understand how receipt of ANC relates to other vital health services along the continuum of care for these populations [11]. If poor women who receive ANC are not returning to the health system or practicing positive health behaviors, this would be a grave missed opportunity to reduce existing health and healthcare access inequities. Alternately, if ANC is shown to be an effective means of engaging and keeping women in the health system, it should receive additional emphasis.

For decades, Latin America had the highest income inequality in the world, but a relative decline in income inequality was seen in the beginning of the twenty-first century through 2010, largely attributable to education, tariff, and trade changes [12, 13]. Still, enormous variation in health coverage and outcomes exists even within the poorest populations [14, 15]. A previous analysis of poor women in Mesoamérica found that education, media exposure, insurance, and indigenous language fluency were significant predictors of in-country variation of ANC uptake [16]. The question remains whether those women who do enter the health system for ANC continue to take part in other interventions in the continuum of care. In this analysis, we examine the relationship between ANC and uptake of related maternal and child health behaviors and services among the poorest women in Guatemala, Honduras, Nicaragua, and the state of Chiapas in Mexico.

## Methods

### Study design and participants

Data for the analysis came from the baseline evaluation of the Salud Mesoamérica Initiative (SMI), a results-based financing program targeting maternal and child health disparities in eight Mesoamérican countries. The methodology of this study has been previously described [17]. In brief, we identified the regions containing the poorest quintile of the population and randomly selected census segments using probability proportional to size of the segment population. We conducted our own census in each of the selected segments and used this information to identify eligible households that contained women of childbearing age, 15 to 49 years, and children under age five. Among eligible households, we selected a random sample of 30 households per visited segment to conduct the full SMI survey.

For the present analysis, we used data from Guatemala, Honduras, Mexico (state of Chiapas), and Nicaragua due to data availability. We used information from three components of the household survey: basic household demographics including assets, expenditure, and languages spoken; a complete birth history and maternal health questionnaire for each eligible woman; and a questionnaire about the health of each child under the age of five. We restricted data to each mother’s youngest child under two years of age so as to minimize recall bias. We linked information about that child’s birth (prenatal, delivery, and postnatal care) to the child health questionnaire using non-identifying IDs.

Baseline surveys were conducted from March 1, 2011 to August 31, 2013 by trained interviewers using computer-assisted personal interviews. Surveys were conducted in Spanish or an indigenous language when appropriate. Data were sent to a secure database at the Institute for Health Metrics and Evaluation for real-time quality assurance monitoring. All data were collected after informed consent was received from the participant; IRB approval did not require parental consent from adolescent mothers. The institutional review boards of the University of Washington, partnering data collection agencies, and the Ministry of Health of each involved country approved the study.

### Predictor of interest: Antenatal care definitions

Women reported whether they received any ANC for their most recent pregnancy and the total number of ANC visits. We additionally recorded the type of provider seen at each visit, except in Guatemala where women reported their usual provider of ANC. We defined skilled ANC as care with a medical doctor or licensed nurse. Reported non-skilled providers included unskilled midwives, auxiliary nurses without a university degree, community health workers, lab technicians, pharmacy assistants, traditional healers, and relatives.

For the present analysis, we used a categorical variable for the number of ANC visits a woman attended as our predictor of interest. The ANC variable contained three levels: no uptake of skilled ANC, at least one but fewer than the WHO-defined “focused ANC” consisting of four skilled visits, and four or more skilled visits. Henceforth, these levels will be referred as no ANC, partial ANC, and complete ANC.

### Outcome definitions

Outcome variables described behaviors and interventions along a continuum of continuity of care subsequent to ANC and fell into two categories: maternal and child.

#### Maternal outcomes

In-facility delivery (IFD) was defined as a woman’s self-report that she gave birth inside of a hospital or clinic. Postpartum care was defined as a woman’s self-report that she received care from a skilled medical professional at least once between seven and 42 days after giving birth. The family planning outcome variable specifically referred to uptake of a modern method of contraception within six weeks postpartum, as is detailed in WHO norms so as to avoid rapid repeat pregnancy, which is traumatic on a woman’s body [18]. Modern forms of contraceptive included female sterilization, male sterilization, IUD, injectables, implants, pill, condom, female condom, diaphragm, sponge/spermicide, and emergency contraception. Immediate breastfeeding was defined as a mother putting her child to her breast within one hour after delivery. Exclusive breastfeeding was determined based on a 24-hr dietary recall for all infants born in the six months prior to the survey, given that WHO recommends exclusive breastfeeding for all children for the first six months.

#### Child outcomes

Postnatal care was based on the mother’s report that a medical professional checked the child within seven days after the birth, excluding visits that happened immediately after birth for IFDs. Babies that were in the neonatal intensive care unit (NICU) or in a facility for more than seven days after birth were excluded from the denominator. Vitamin A supplement uptake was based upon the mother’s report that her child received a dosage of vitamin A in the last six months, as norms require that children receive oral supplements twice a year until the age of five. Vaccine compliance was based upon each individual country’s norms for immunization by age. To be compliant, a child needed to receive every recommended vaccine based upon the information recorded on his or her immunization card in accordance with the country-specific immunization scheme.

### Analysis

We used logistic regressions to assess the associations between uptake of ANC and subsequent uptake of services and healthy behaviors. Each outcome was binary. We accounted for survey weights and clustering by segment using the survey commands in Stata 13.1. We stratified on country because the relationship between antenatal care and the selected outcomes likely differs based upon the health services conditions, as well as the social and political context.

We controlled for a range of demographic, socioeconomic, and health-related covariates that were significantly associated with receiving complete ANC for any country in a previous analysis of this data [16]. Specifically, these covariates were maternal education, maternal age, parity, household expenditure, exposure to media in the last week (newspaper, radio, or television), whether or not the pregnancy was desired, and a categorical indicator of travel time to the self-reported usual health facility in minutes. If the usual health facility was missing, we used the closest health facility. In Guatemala, Mexico, and Nicaragua we included in the models an indicator of whether or not anyone in the household spoke an indigenous language. Health insurance status in Guatemala was considered as a covariate but ultimately not included because it was not statistically associated with ANC (*p* > 0.05). Prospera (formerly Oportunidades) enrollment was included for Mexico. In addition to these predictive covariates from Dansereau et al., we included in the final model the following binary indicators that were correlated with at least one of our outcomes (*p* < =0.05): whether or not the child was a firstborn, whether the mother was currently in a marriage or union, whether the mother had a previous pregnancy that did not result in a live birth (abortion, stillbirth, or miscarriage), whether the mother had received counseling from a community health worker in the past month, and an indicator of urbanicity. We also adjusted for a household asset score. Our asset score was calculated by giving a household one point for each asset from a list of country-specific assets (e.g., television, mobile phone, refrigerator, and guitar). Within each country, the sum of the number of assets each household possessed was divided by the number of possible assets.

We controlled for multiple comparisons using a permutation test, a method through which to derive the sampling distribution and, under the null hypothesis, test the probability of finding significance at least as large as the observed estimate. We permuted the dataset 1000 times to simulate a distribution of test statistics under the null hypothesis that ANC has no effect on a woman’s likelihood of remaining in the continuum of care. Thus, our reported *p*-values represent the proportion of permuted datasets out of the 1000 that give a test statistic greater than or equal to the nominal test statistic from the observed data.

### Ethics

Data were collected after informed consent was confirmed from all participants, including adolescent mothers. The study received approval from the institutional review board (IRB) from the University of Washington, partnering data collection agencies, and the Ministry of Health in each country. The investigators did not receive identifiable data and survey components were linked using non-identifying IDs.

### Role of the funding source

The funders of this study had no role in the present analysis. The corresponding author had full access to the data and final responsibility for decision to submit for publication.

## Results

Table 1 describes the demographic characteristics of our sample in each country. Women reported 7125 births in the past two years in Guatemala, Honduras, Mexico (state of Chiapas), and Nicaragua. Of these births, we were able to link 5045 (70.8%) to a child survey based upon unique household ID, woman ID, and child ID. Ninety-six percent of these woman/child linkages (*N* = 4844) contained a full set of covariates and were retained for the final analysis.

**Table 1:** Demographic, household, and health characteristics of sample women

	Guatemala	Honduras	Mexico	Nicaragua	All
N	1445	846	1791	762	4844
Education					
None	28.3%	5.7%	14.5%	10.1%	13.3%
Primary/literacy course	52.6%	70.8%	52.9%	46.1%	52.9%
Post-primary	19.1%	23.5%	32.6%	43.8%	33.8%
Age category					
< 20 years	18.4%	17.6%	15.4%	18.6%	16.7%
20 to 34 years	64.3%	67.6%	70.2%	69.1%	69.2%
> =35 years	17.3%	14.8%	14.4%	12.3%	14.1%
Occupation					
Employed & working	4.4%	6.7%	4.4%	11.5%	6.6%
Homemaker	92.9%	90.7%	94.1%	85.7%	91.4%
Other	2.8%	2.6%	1.5%	2.9%	2.1%
Married/union	86.8%	80.9%	92.8%	76.6%	86.7%
Average parity	3.06	2.65	3.10	2.43	2.87
Child’s age					
< 6 months	28.0%	27.6%	26.5%	25.6%	26.5%
6 to 12 months	27.9%	26.5%	28.6%	29.7%	28.6%
13 to 24 months	44.1%	45.8%	44.9%	44.7%	44.9%
Female child	50.6%	50.9%	49.6%	50.8%	50.1%
Previous obstetric complication not resulting in live birth	6.7%	9.9%	6.2%	8.7%	7.3%
Wanted pregnancy	84.7%	73.1%	79.4%	68.2%	76.0%
Any media exposure (in the last week)	67.5%	80.2%	66.3%	87.9%	73.7%
Indigenous (language spoken in household)	73.2%	0.1%	70.1%	16.2%	48.5%
Contact with CHW (in last month)	6.8%	11.3%	19.0%	2.0%	12.7%
Has insurance	11.4%	1.0%	81.4%	4.2%	47.1%
Urban	16.8%	11.8%	36.1%	33.7%	31.6%
Travel time					
< 15 min.	31.5%	32.1%	34.3%	23.2%	30.9%
15 to 30 min.	27.4%	26.1%	26.1%	26.8%	26.4%
30 to 60 min.	21.4%	19.8%	23.8%	20.9%	22.4%
60+ min.	19.7%	22.0%	15.7%	29.1%	20.3%
Average assets score	0.24	0.25	0.22	0.23	0.23

The large majority of women in our dataset were married or in a union (86.7%) and homemakers (91.4%). Most women had received primary education or a literacy course, and only 33.8% received post-primary education. Only 16.7% of mothers were under 20 years of age. Those living in urban locations ranged from 11.8% in Honduras to 36.1% in Mexico. The proportion of women reporting having experienced a stillbirth, abortion, or miscarriage in the past was less than 10% in all countries. About three-fourths of women reported that they wanted to conceive the child (76.0%).

**Fig. 1:**
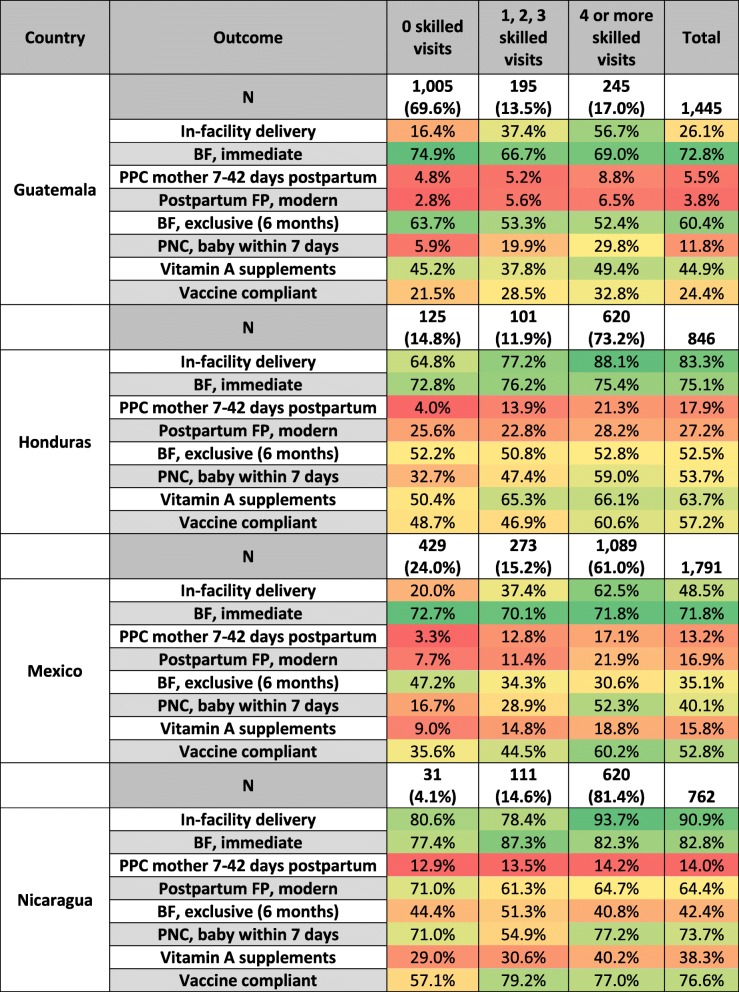
Heatmap showing the uptake of each outcome variable considered

Although about two-thirds of all women received at least one skilled ANC visit during their most recent pregnancy in the last two years (67.2%), this varied greatly by country. Only 4% of women did not attend any skilled ANC in Nicaragua, while 70% did not attend skilled care in Guatemala, as shown in Fig. 1. The number receiving the WHO-recommended four visits ranged from 17.0% in Guatemala to 81.4% in Nicaragua.

Outcome prevalence is also shown in Fig. 1. Reporting of IFDs ranged from 26.1% in Guatemala to 90.9% in Nicaragua. Around three-quarters of women reported breastfeeding immediately in each country. Mothers reported receiving postpartum care for themselves at rates ranging from 5.5% in Guatemala to 17.9% in Honduras, although postnatal care for the baby was higher, ranging from 11.8% in Guatemala to 73.7% in Nicaragua. Use of a modern family planning method within six weeks postpartum ranged from only 3.8% in Guatemala to 64.4% in Nicaragua. Exclusive breastfeeding for the first six months ranged from 35.1% in Mexico to 60.4% in Guatemala. Receipt of a vitamin A supplement ranged from 15.8% in Mexico to 63.7% in Honduras, and vaccine compliance for age ranged from 24.4% in Guatemala to 76.6% in Nicaragua.

Regression results for maternal outcomes are shown in Table 2, and all odds ratios are adjusted for the covariates described above. Results of multivariable models for each outcome are described below.

**Table 2:** Adjusted regression results for maternal outcomes

	ANC	Guatemala^b^		Honduras		Mexico^c^		Nicaragua^d^	
		OR	p	OR	p	OR	p	OR	p
In-facility delivery	1–3 ANC visits	**3.16** ^**a**^	**	**2.50**	**	1.56		**2.49**	**
		**(2.29–4.36)**		**(1.65–3.79)**		(0.77–3.17)		**(1.58–3.93)**	
	4 or more ANC visits	**7.64**	**	**5.26**	**	2.60		**4.99**	**
		**(5.87–9.95)**		**(3.61–7.66)**		(1.42–4.77)		**(3.41–7.30)**	
Immediate breastfeeding	1–3 ANC visits	1.18		0.64		1.05		0.98	
		(0.87–1.60)		(0.41–0.98)		(0.55–2.00)		(0.65–1.47)	
	4 or more ANC visits	**1.37**	*	0.72		1.25		1.18	
		**(1.08–1.74)**		(0.50–1.05)		(0.84–1.85)		(0.85–1.64)	
Postpartum care for mother (7–42 days post delivery)	1–3 ANC visits	0.88		**3.97**	**	**4.18**	**	1.07	
		(0.34–2.26)		**(1.39–11.30)**		**(1.84–9.51)**		(0.26–4.41)	
	4 or more ANC visits	1.54		**5.57**	**	**6.31**	**	1.15	
		(0.73–3.26)		**(2.11–14.70)**		**(3.48–11.45)**		(0.31–4.30)	
Postpartum modern family planning (within 6 weeks of delivery)	1–3 ANC visits	**3.08**	**	1.99		**1.71**	*	**1.71**	*
		**(2.13–4.45)**		(0.85–4.66)		**(1.04–2.80)**		**(1.04–2.80)**	
	4 or more ANC visits	**4.67**	**	**2.31**	*	**2.21**	*	**2.21**	**
		**(3.48–6.26)**		**(0.97–5.54)**		**(1.45–3.37)**		**(1.45–3.37)**	
Exclusive breastfeeding (6 mos.)	1–3 ANC visits	0.78		0.61		1.03		**0.64**	*
		(0.59–1.04)		(0.36–1.04)		(0.56–1.91)		**(0.45–0.92)**	
	4 or more ANC visits	**0.64**	**	**0.55**	*	1.24		0.69	
		**(0.51–0.81)**		**(0.34–0.90)**		(0.74–2.07)		(0.48–0.99)	

*p < 0.05; **p < 0.01

^a^All regressions controlled for: maternal education, maternal age category, maternal occupation, parity, marriage status, previous problematic pregnancies, whether the pregnancy was desired, asset score, household expenditure quintile, media exposure, travel time, community health worker visits in the last month, and urbanicity

^b^In Guatemala, we additionally controlled for indigeneity

^c^In Mexico, we additionally controlled for indigeneity and opportunidades participation

^d^In Nicaragua, we additionally controlled for indigeneity

### In-facility delivery

About half of Guatemalan mothers with full ANC delivered their baby in a health facility, compared to only 16.4% of those who did not attend ANC. Partial and complete ANC in Guatemala was associated with significantly higher odds of IFD compared to those who did not receive any ANC (Partial: OR = 2.49 [95% CI: 1.64–3.78]; Complete: OR = 5.28 [95% CI: 3.62–7.69]). The same trend emerged in Mexico (Partial: OR = 2.49 [95% CI: 1.58–3.92]; Full: OR = 5.00 [95% CI: 3.41–7.32]) and in Honduras for those mothers who received complete ANC (OR = 2.60 [95% CI: 1.42–4.78]). There was no significant difference in Nicaragua.

### Immediate breastfeeding

In all four countries and across all ANC levels, reports of immediate breastfeeding were high, ranging from 66.7% in Guatemala to 87.3% in Nicaragua. In all four countries, receipt of partial or complete ANC was not significantly associated with higher odds of immediate breastfeeding relative to those who did not attend ANC, though there was a near-significant negative trend amongst Guatemalan women receiving partial ANC (OR = 0.64 [95% CI: 0.41–0.99]).

### Postpartum care for mother (7–42 days post-delivery)

Uptake of postpartum care was generally low in all four countries, ranging from 5.5% of all women in Guatemala to 17.9% in Honduras. Relative to mothers who did not receive ANC, Honduran and Mexican mothers with complete uptake had nearly six times greater odds of receiving postpartum care than those who did not receive ANC (OR = 5.78 [95% CI: 2.19–15.24]; OR = 5.79 [95% CI: 2.98–11.24], respectively). In Guatemala and Nicaragua, there was no significant difference in uptake of postpartum care amongst women who received no, partial, or complete ANC.

### Postpartum modern family planning (within six weeks of delivery)

Postpartum family planning was especially low in Guatemala – ranging from 2.8% among women with no care to 6.5% amongst women with complete care – but Guatemalan women who received complete ANC had increased odds of beginning modern family planning methods within six weeks of delivery (OR = 2.32 [95% CI: 0.97–5.55]). In Honduras and Mexico, approximately one-quarter of women reported use within the first six weeks, with no significant difference across ANC levels. In Nicaragua, uptake of family planning methods was 71.0% for women with no skilled visits and 64.7% for women with complete ANC, with no significant differences across ANC levels.

### Exclusive breastfeeding for children over six months

Mothers in Guatemala who received complete ANC had significantly lower odds of exclusive breastfeeding for the entirety of the first six months (OR = 0.56 [95% CI: 0.34–0.90]). While 60.4% of women who received full ANC exclusively breastfed for six months, 63.7% of women who received no ANC breastfed exclusively. Those in Chiapas also had lower odds of exclusive breastfeeding if they received partial or complete ANC relative to mothers who received no ANC (Partial: OR = 0.64, [95% CI: 0.45–0.92]; Complete: OR = 0.69 [95% CI: 0.48–0.99]). 47.2% of mothers in Chiapas with no ANC reported exclusive breastfeeding, while only 30.6% of those with complete ANC reported the same. There was no significant difference in odds of exclusive breastfeeding for at least six months in Nicaragua and Honduras.

Results for the child outcomes are displayed in Table 3, using the final model which controlled for all variables described above.

**Table 3:** Adjusted regression results for child outcomes

	ANC	Guatemala^b^		Honduras		Mexico^c^		Nicaragua^d^	
		OR	p	OR	p	OR	OR	p	OR
Postnatal care for baby within 7 days	1–3 ANC visits	**2.43** ^**a**^	**	**3.33**	**	1.57		**1.86**	**
		**(1.81–3.26)**		**(2.05–5.40)**		(0.84–2.94)		**(1.22–2.85)**	
	4 or more ANC visits	**6.12**	**	**4.81**	**	2.14		**4.01**	**
		**(4.68–7.99)**		**(3.20–7.21)**		(1.26–3.63)		**(2.77–5.81)**	
Vitamin A supplements	1–3 ANC visits	0.99		0.76		**1.96**	*	1.40	
		(0.74–1.33)		(0.54–1.07)		**(1.13–3.41)**		(0.82–2.38)	
	4 or more ANC visits	**1.59**	**	1.30		**2.09**	*	**1.85**	**
		**(1.27–1.99)**		(0.92–1.83)		**(1.47–2.99)**		**(1.17–2.93)**	
Vaccine compliant	1–3 ANC visits	**2.37**	**	1.09		0.91		1.49	
		**(1.69–3.31)**		(0.67–1.79)		(0.51–1.63)		(0.94–2.35)	
	4 or more ANC visits	**3.41**	**	1.34		1.15		**2.70**	**
		**(2.66–4.37)**		(0.90–2.01)		(0.75–1.76)		**(1.87–3.89)**	

* *p* < 0.05; **p < 0.01

^a^All regressions controlled for: maternal education, maternal age category, maternal occupation, parity, marriage status, previous problematic pregnancies, whether the pregnancy was desired, asset score, household expenditure quintile, media exposure, travel time, community health worker visits in the last month, and urbanicity. Models for vitamin A supplements, vaccine compliancy for age, and postnatal care for the baby additionally included controls for the child’s age in months at the time of the survey and the sex of the child

^b^In Guatemala, we additionally controlled for indigeneity

^c^In Mexico, we additionally controlled for indigeneity and opportunidades participation

^d^In Nicaragua, we additionally controlled for indigeneity

### Postnatal care for babies within seven days

Babies born in Guatemala had increased odds of receiving postnatal care if their mothers received partial ANC (OR = 3.30 [95% CI: 2.04–5.35]), and further increased odds if their mothers received complete ANC (OR = 4.82 [95% CI: 3.21–7.23]). Uptake ranged from 5.9 to 29.8% across levels of ANC. The same was true in Mexico, where uptake ranged from 16.7 to 52.3%. (Partial: OR = 1.87 [95% CI: 1.22–2.85]; Complete: OR = 4.02 [95% CI: 2.77–5.82]). In Honduras, babies whose mothers received no or partial ANC did not have statistically different odds of receiving postnatal care within the first week after birth, but those whose mothers received complete ANC did have increased odds compared to those with no ANC (OR = 2.14 [95% CI: 1.26–3.64]). There was no difference in Nicaragua, where the majority of babies received care regardless of ANC level.

### Vitamin a supplements

Mothers in Honduras had the highest reports of vitamin A supplement use, ranging from 50.4 to 66.1% across ANC levels. Odds of using vitamin A supplements if the mother received full ANC were twofold those of their counterparts who received no ANC (Partial: OR = 1.96 [95% CI: 1.13–3.42]; Complete: OR = 2.10 [95% CI: 1.47–3.00]). In Guatemala, Mexico, and Nicaragua, uptake was lower and there was no significant difference in odds of receipt of vitamin A supplements in the last six months. Overall uptake was 44.9, 15.8, and 38.3% for Guatemala, Mexico, and Nicaragua, respectively.

### Vaccine compliance

Nicaraguan children had the highest rates of vaccine compliance overall (76.6%). Nicaraguan mothers who received partial or complete ANC had greater odds of having vaccine compliant children (Partial: OR = 4.75 [95% CI: 1.82–12.42]; Complete: OR = 2.64 [95% CI: 1.30–5.36]). In Guatemala, Honduras, and Mexico, there was no significant difference; overall rates were 24.4, 57.2, and 52.8%, respectively.

## Discussion

This analysis is the first to look specifically at the relationship between uptake of ANC and continued participation in the continuum of care in Mesoamérica. The WHO claims that educational components of ANC are intended to keep women in the system, and national clinical guidelines reflect this message; nevertheless, evidence that ANC succeeds in reducing dropout from the continuum of care is incomplete [19]. This study adds to this body of literature, with a particular focus on the most impoverished populations in Mesoamérica. Our results demonstrate that uptake of the WHO-defined “focused ANC” of four ANC visits is associated with increased uptake of services in some poor populations in Mesoamérica, but that this relationship varies greatly across countries. Indeed, our study comes in the context of great efforts toward universal health coverage in Latin America, including health system reforms in Nicaragua and El Salvador, the Accelerated Reduction of Maternal and Child Mortality (RAMNI) program in Honduras, and Prospera and Seguro Popular in Mexico. Nevertheless, our study highlights the need for continued and varied efforts in these populations to increase both the uptake and the effectiveness of ANC in encouraging positive and lasting effects on women’s uptake of health care services.

Findings highlight substantial differences in the coverage of ANC across countries in the region, with skilled ANC coverage highest in Nicaragua and lowest in Guatemala. These differences may be due to differential coverage of the health system, but also due to the implementation of significant outreach activities, which are in place in Nicaragua [20, 21].

Though IFD rates and postnatal care uptake were low overall amongst Guatemalan women, a significant effect was found amongst women who received partial and complete ANC. This finding may suggest that ANC has the potential to drive change in women’s decisions to return to the facility and seek more care, although it is also possible that some women use more health services overall and attend ANC and IFD more frequently. Regardless, postnatal care for the child should serve as an opportunity to remind women to return to the facility for postpartum care for the mother 7–42 days after birth, yet postpartum care uptake is still low and thus a critical opportunity for improvement.

In Honduras and Mexico, ANC uptake was significantly associated with uptake of postnatal and postpartum care, two services with low uptake rates overall. The two possible explanations mentioned above could play a role in this case. Postpartum family planning methods have similarly low uptake rates; however, there was no significant variation in uptake of postpartum family planning methods among different levels of ANC, and even a non-significant but negative relationship with ANC level in Honduras, Mexico, and Nicaragua. Publicly sponsored family planning has been offered in Mexico since 1977, and family planning education is in the national antenatal care guidelines. A study conducted at the national level in Mexico found that quality of ANC, but not timing (first trimester of pregnancy), was associated with postpartum family planning [22]. The fact that these poor populations are not benefiting from these services, even among women receiving the WHO-recommended four ANC visits, is concerning and suggests there may be cultural or availability factors at play that policymakers and clinicians must seek to understand [23]. Family planning promotion is critical for these vulnerable populations. Unwanted pregnancies during breastfeeding and prior to the return of a woman’s menses are serious health risks in low-resource settings [24–26]. With proper counseling at ANC, perhaps women can be empowered to space their births and thereby have healthier pregnancies and deliveries.

Honduran women who received ANC were more likely to report vitamin A receipt for their child, likely due to the fact that conditional cash transfer programs exist in Honduras that both require ANC for pregnant women enrolled in the program and provide vitamin A supplements for children. It is concerning that this same effect did not exist with vaccine compliance, another component of the conditional cash transfer program [27]. Resources should address this gap.

Though effect size is small, it is worth mentioning the concerning result that Guatemalan women receiving partial or complete ANC and Mexican women receiving partial ANC were less likely to exclusively breastfeed compared to their counterparts receiving no ANC. While we did adjust for possible confounders, this counterintuitive relationship is likely due to already high rates of breastfeeding in these populations, as well as additional residual confounding. Inequalities exist even within the poorest populations, and higher status, more educated women are less likely to breastfeed, perhaps because they work or can afford formula. A previous analysis using this data documented this concerning relationship [28]. Given that ANC did not significantly increase breastfeeding behaviors in any of the study countries, however, it may be that breastfeeding behaviors are determined more by cultural influences than by the instructions received at a health facility. ANC might have greater potential to influence uptake of services that rely on health facilities, rather than autonomous behaviors like breastfeeding.

Because Nicaragua had the smallest sample size and the highest uptake of services across ANC levels and of ANC itself, it had the least room for improvement. Nicaragua has an important community platform paired with strong community outreach campaigns to sensitize the population. In spite of the fact that ANC is well accepted and service uptake is high, ANC level was still significantly associated with vaccine compliance, likely due to the success of frequent outreach activities for maternal and child health.

### Limitations

Our results should be interpreted with some limitations in mind. First, we rely on self-reported data, and it is possible that women might misreport the number of ANC visits they attended, the provider they saw, or their uptake of services. However, we minimized recall bias by limiting the analysis to information about a woman’s most recent pregnancy in the last two years. Second, our study only considered skilled ANC, as is recommended in country and WHO norms, and ANC was defined as skilled if the woman saw a doctor or registered nurse. The difference between receiving ANC by non-skilled versus skilled personnel, as reported by the woman, was only 1.7% in Nicaragua, 11.0% in Honduras, and 18.7% in Mexico (most frequently due to the use of unskilled midwives or traditional birth attendants). This difference was greatest in Guatemala at 53.5%, largely due to the popularity of auxiliary nurses. Nevertheless, if non-skilled providers are giving adequate care, this limitation would bias our results towards the null; thus, our results are conservative and any significant results demonstrate the particular potential of *skilled* care. Future analyses should consider the effectiveness of unskilled antenatal care in these populations. Third, the present analysis looks exclusively at ANC in terms of quantity, and neglects to consider quality or content of visits. Future research should further examine the potential for more nuanced information about what happens at ANC in order to help explain the mechanisms behind the mixed associations with participation in the continuum of care. Finally, the present observational study demonstrates association, not causation, though strong relationships persist even when controlling for a wide variety of possible confounders. A cohort analysis would be invaluable in corroborating our results and understanding the multiple possible effects examined in the present study; however, one considerable strength of our study is its reliance on the most up-to-date sampling frame possible, in contrast to studies that develop sampling frames based on censuses which may be outdated.

## Conclusions

Despite continued financial efforts, multi-tier interventions, and policy reform toward improved maternal and child health in Guatemala, Honduras, Nicaragua, and Chiapas, Mexico, there remains significant need for improvement. Indeed, ANC uptake should be a priority, but efforts to increase the uptake of ANC miss their full potential if the benefits end after a woman leaves her appointment. Successful ANC is care that not only screens for problematic pregnancies, but goes further and educates women about how and when to access the health system for critical care when and after the child is born.

Our findings suggest that countries need to strengthen their work at the community level to track pregnant women and encourage them to attend all four recommended ANC visits, as well as encourage behaviors that were not associated with ANC, such as exclusive breastfeeding. Moreover, countries must intervene at the facility level in order to ensure satisfaction and encourage women who attend one visit to return for a second, third, and fourth ANC visit. A higher number of ANC visits will give more opportunities to reiterate educational messages and affirm the benefits that can be reaped by continued interaction with the health system. This intervention could take the form of follow-up reminders, using new technologies like text messages to a woman’s cell phone or the phone of a family member.

Although this study explores the association between ANC and the uptake of interventions in the continuum of care, many questions remain. Further research should consider why there is no association between ANC and immediate breastfeeding. Furthermore, WHO recommendations about antenatal care detail quality of care expectations, about which we have limited information. The characteristics of the ANC, as well as the messaging received at the ANC, might further explain its effectiveness in promoting health behaviors. Some behaviors may be inherently easier to modify than others, which is a consideration that behavioral research could add to this discussion.

Our results were largely country-dependent. Countries can and should observe and emulate the most effective ANC practices among similar populations in neighboring countries in order to prevent missed opportunities to keep the most vulnerable women and children in the health system. Indeed, the Salud Mesoamérica Initiative could be adopted to create a network to share valuable information within and between countries in the region.

## Abbreviations

ANC: Antenatal Care
CI: Confidence Interval
OR: Odds Ratio
SMI: Salud Mesoamérica Initiative
WHO: World Health Organization
